# Can an acute high-grade acromioclavicular joint separation be reduced and stabilized without surgery? A surgeon’s experience

**DOI:** 10.1007/s00402-020-03630-0

**Published:** 2020-10-27

**Authors:** Tazio Maleitzke, Nina Maziak, Fabian Plachel, Tobias Winkler, Philipp Moroder

**Affiliations:** 1 Center for Musculoskeletal Surgery, Charité–Universitätsmedizin Berlin, Augustenburger Platz 1, 13353 Berlin, Germany; 2 Julius Wolff Institute, Charité–Universitätsmedizin Berlin, Augustenburger Platz 1, 13353 Berlin, Germany; 3 Berlin Institute of Health Center for Regenerative Therapies, Charité–Universitätsmedizin Berlin, Augustenburger Platz 1, 13353 Berlin, Germany; 4 Berlin Institute of Health (BIH), 10178 Berlin, Germany

**Keywords:** Acromioclavicular joint separation, Acromioclavicular joint dislocation, Conservative therapy, Shoulder injury, Rockwood, Tossy

## Abstract

**Introduction:**

While the management of Rockwood type III injuries is still a topic of debate, high-grade Rockwood type V injuries are mostly treated surgically, to anatomically reduce the acromioclavicular (AC) joint and to restore functionality. In this case report, we present a method for non-operative reduction and stabilization of a high-grade AC joint injury.

**Case:**

A 31-year-old male orthopaedic resident sustained a Rockwood type V injury during a snowboarding accident. His AC joint was reduced and stabilized with an AC joint brace for six weeks. The brace provided active clavicle depression and humeral elevation. After removal of the brace the AC joint showed a nearly anatomic reduction. Six-month follow-up weighted X-ray views showed an AC joint which had healed in a Rockwood type II position and the patient returned to full pre-injury function with a satisfying cosmetic appearance.

**Conclusion:**

Non-operative reduction and stabilization of high-grade AC joint separations seems to be a valuable treatment option. A “closed reduction and external fixation” approach with the aid of a dedicated AC joint brace can reduce the AC joint and keep it in place until ligamentous consolidation occurs, thus improving AC joint stability and cosmetic appearance without surgical intervention.

## Introduction

AC joint separations account for 4–12% of all shoulder injuries and mainly affect male athletes that sustain a direct impact trauma to the shoulder [1]. The original Rockwood classification from 1984 is still commonly used to identify the severity of the ligamentous injury and to determine the therapeutic regimen [2]. Depending on the type of injury, treatment of AC joint separations ranges from conservative immobilization of the shoulder for low-grade injuries to surgical reconstruction of the AC joint for high-grade injuries [3, 4].

Usually, Rockwood type I and II injuries, where the coracoclavicular (CC) ligaments are still intact, are treated conservatively with a shoulder sling for a few days, ice, analgesia and early pain-adapted physiotherapy [4]. While it is generally accepted to treat Rockwood type I and II injuries conservatively, the treatment of Rockwood type III injuries remains controversial and a topic of debate. Several systematic reviews and meta-analyses could not provide conclusive evidence in favour of surgical care [5–8]. Therefore, treatment decisions for Rockwood type III injuries are mostly made on a case-by-case basis [9].

Rockwood type IV–VI injuries are usually treated surgically with a variety of techniques aiming at anatomical reduction and realignment of clavicle and acromion [3]. Reports of conservatively treated Rockwood V injuries are scarce. Yet, a retrospective case series of 18 patients, showed a 61% return-to-duty rate in soldiers who sustained a Rockwood type V injury and who were treated conservatively [10].

The literature describes various conservative methods to treat AC joint separations, ranging from the original spica cast method described by Tossy et al. in 1963 [11] to the nowadays more common shoulder immobilization in a cloth sling [12–14] and isolated attempts to achieve clavicle depression with Leukotape [15]. Most experts combine shoulder immobilization for a few days with physiotherapy, yet no consensus exists regarding conservative treatment algorithms and immobilization devices for the post-injury phase. Typically, early functional training with no reduction or external stabilization is attempted when non-operative treatment is pursued [4].

The main advantage of restoring the AC joint’s anatomic integrity lays in the possibility for the capsule to heal through scaring. If the AC joint is not reduced, scaring around the injured tissue may still occur, but the capsule cannot heal. The position that the clavicle is immobilized in will therefore determine the position in which the injury will heal. Neglected reduction in conservatively treated high-grade AC joint separations often leads to an elevated and prominent distal clavicle under the skin. Many patients are bothered by the cosmetic outcome and function might be impaired in some patients due to a remaining instability after conservative treatment [8].

All slings and braces, currently used in AC joint separation therapy, can provide temporary immobilization but do not address the separation of the joint itself. There are no reports of devices that provide a permanent clavicle depression and humeral elevation to reduce and realign the AC joint.

In this case report, we present a method for non-operative reduction and stabilization of a high-grade AC joint injury, using an AC joint brace for six weeks in combination with a restrictive physiotherapy program.

## Case report

We report the case of a 31-year-old male orthopaedic resident, who presented to our emergency department after a snowboarding accident. On the same morning the patient fell off a rail obstacle in a snowboard park onto his left shoulder. After the direct blast to the shoulder, the patient felt a sudden separation of the AC joint.

**Fig. 1:**
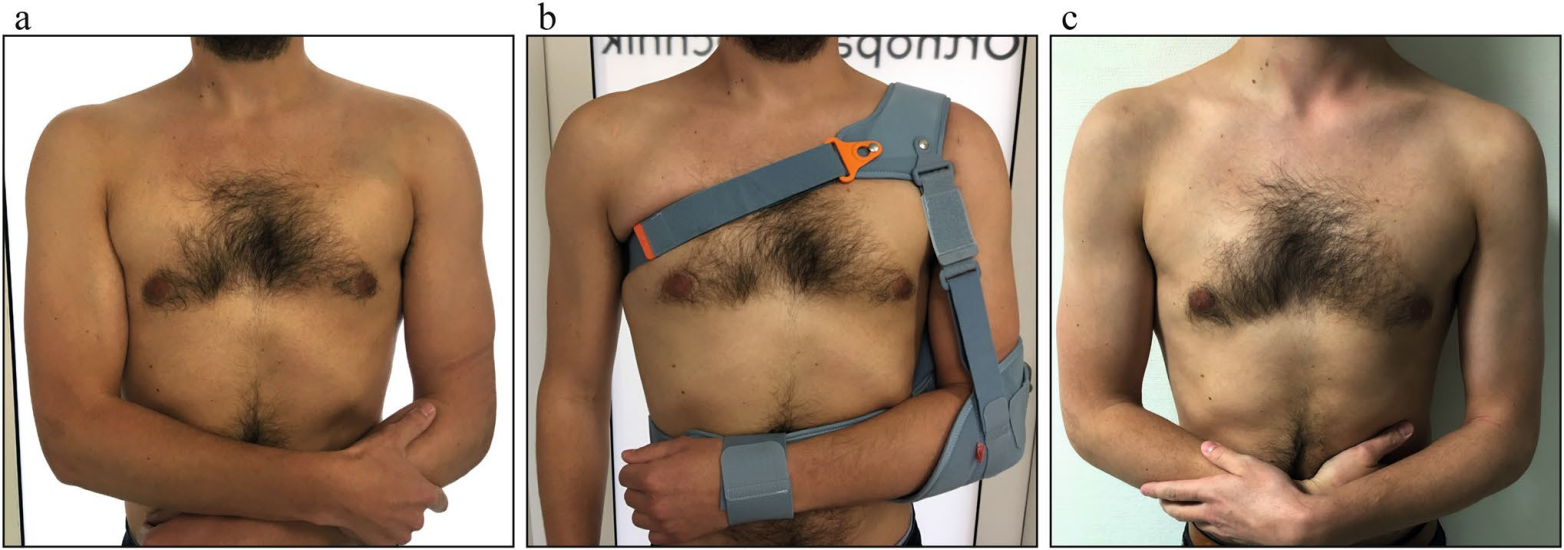
Clinical presentation at different follow-up appointments. **a** Initial presentation of the patient with only a mild elevation of the left distal clavicle on the day of injury. **b** Application of the AC joint brace one day after the injury. **c** Clinical presentation of the patient six weeks after the injury with no signs of an elevated distal clavicle

**Fig. 2:**
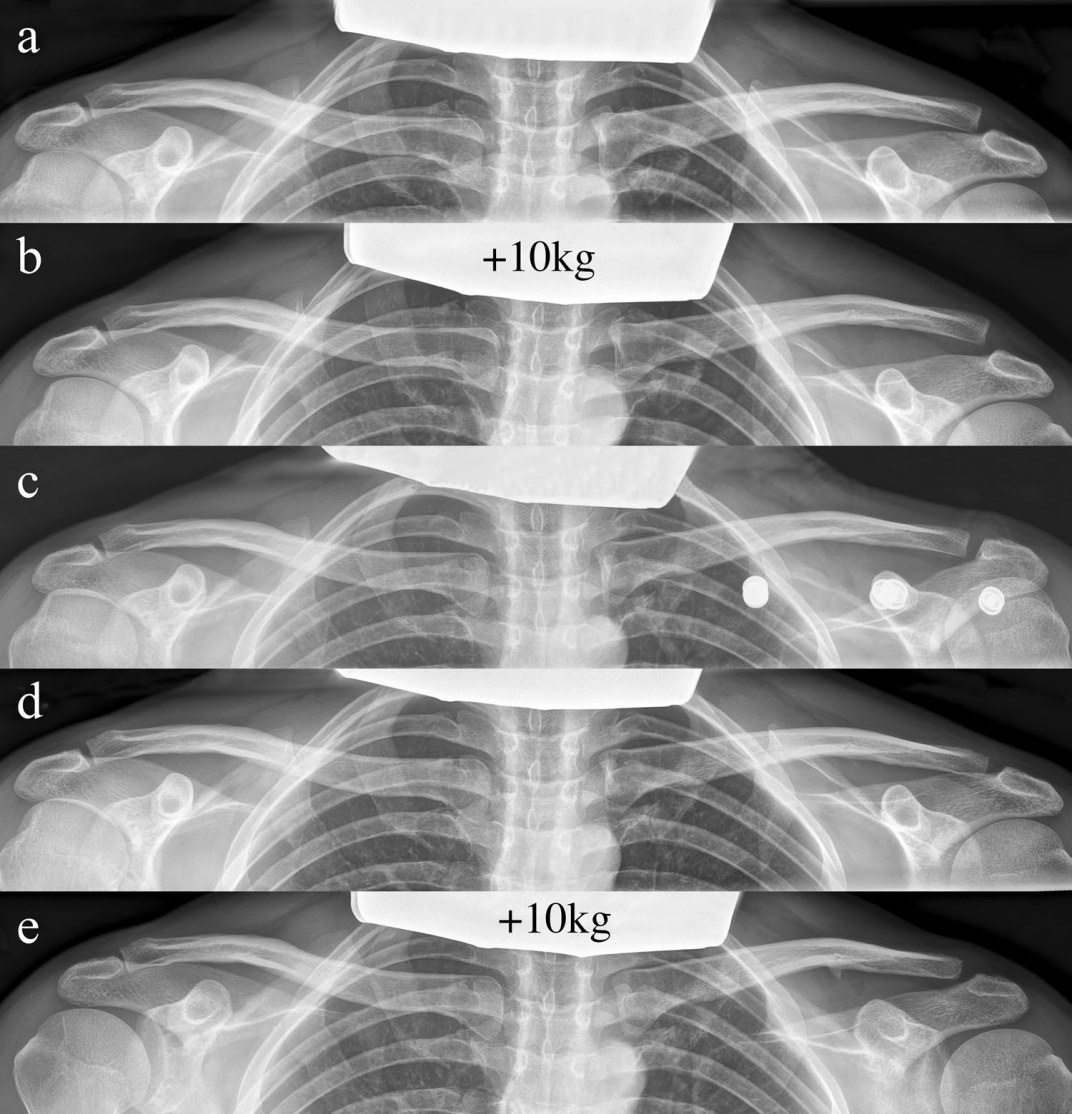
X-ray views at consecutive follow-up appointments. **a** Unweighted bilateral AP X-ray view of clavicles obtained on the day of injury. A Rockwood type III AC joint separation on the left side is evident. **b** Weighted bilateral AP X-ray view of clavicles obtained on the day of injury. 10 kg weights were attached to each wrist and unmasked a Rockwood type V AC joint separation with a CC distance of more than 100% compared to the contralateral healthy side (19 mm vs. 8 mm). **c** Bilateral AP X-ray view of clavicles with the AC joint brace worn on the left side. An anatomic reduction of the left AC joint is visible alongside the projection of three metal buttons just under the left clavicle, stemming from the shoulder pad of the AC joint brace that covers and actively depresses the clavicle. **d** Unweighted bilateral AP X-ray view of clavicles six weeks after conservative treatment. An anatomic alignment of the left AC joint, comparable to the healthy contralateral side, is evident. **e** Weighted bilateral AP X-ray view of clavicles obtained at the six-month follow-up. 10 kg weights attached to each wrist show a Rockwood type II AC joint separation with a mildly elevated left clavicle and a small ossification below the middle third of the clavicle

**Fig. 3:**
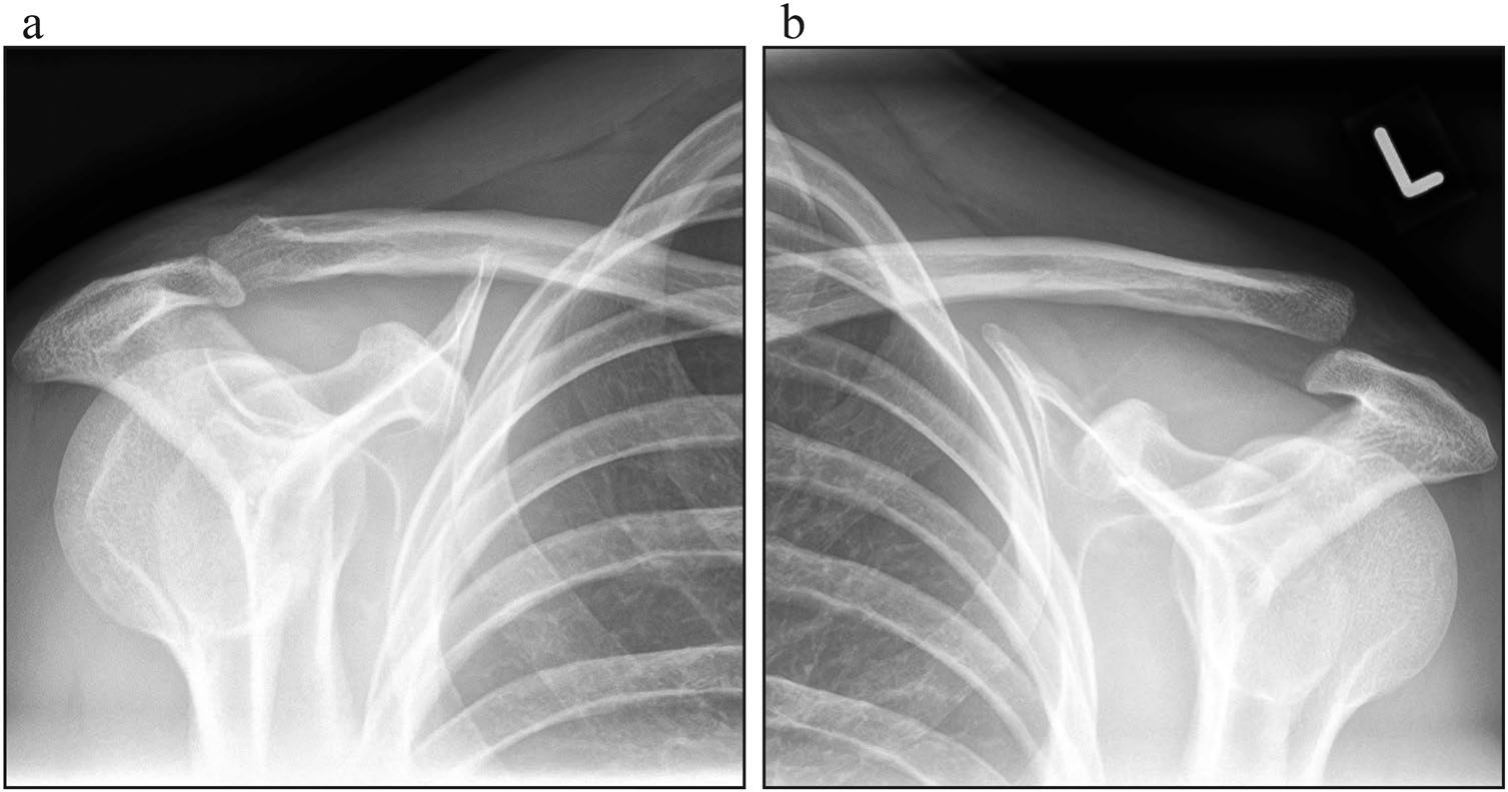
Bilateral Alexander X-ray views. **a** Unweighted bilateral AP axial oblique Alexander X-ray views obtained of the healthy right and **b** injured left AC joint on the day of injury

**Fig. 4:**
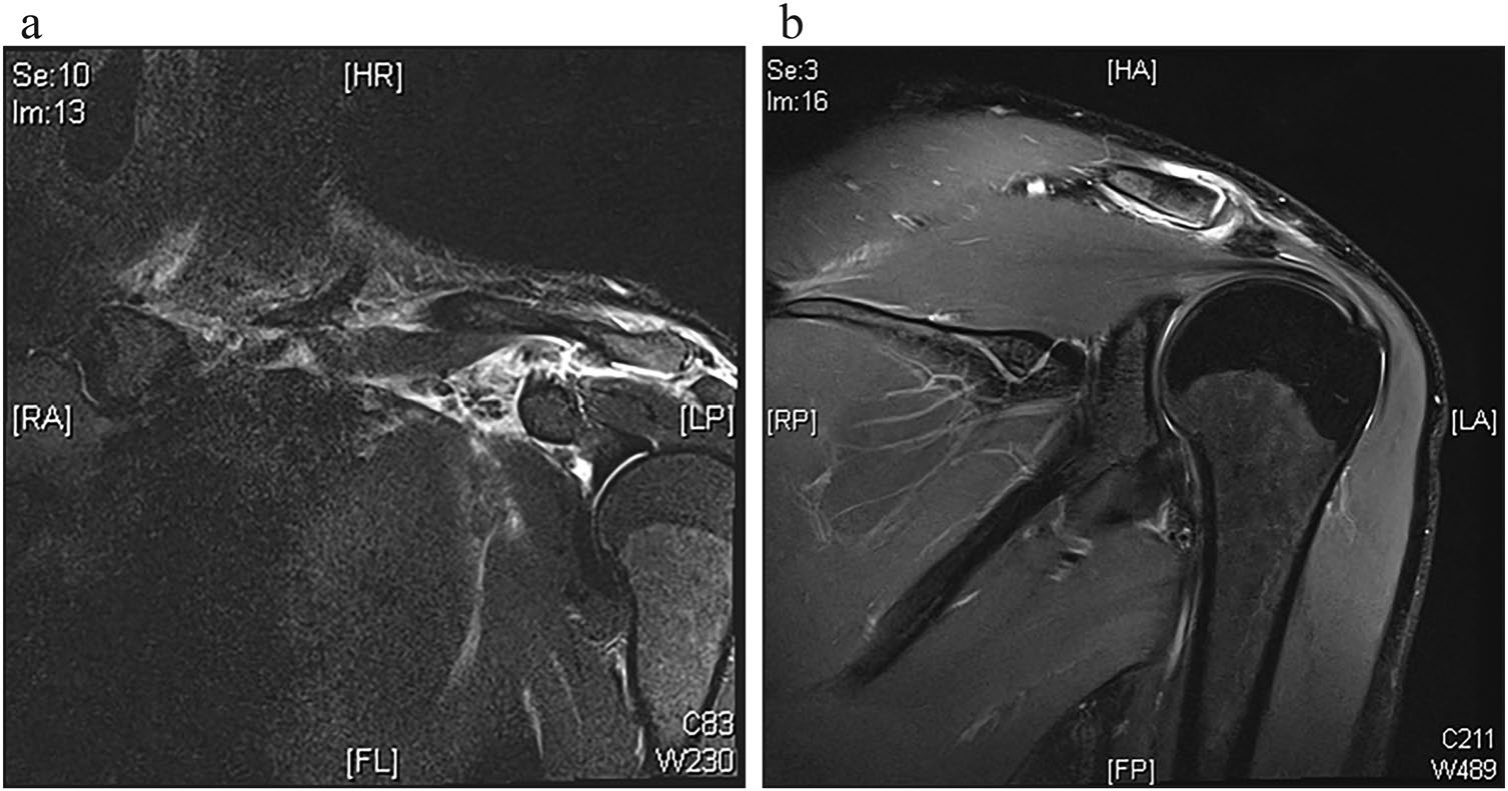
Coronal MRI of the left shoulder region one day after the injury. **a** A total rupture of the AC and CC ligaments with a concomitant edema surrounding the AC joint, **b** alongside a ruptured AC capsule is visible

Upon admission, the patient underwent clinical examination revealing a moderate piano key sign (Fig. 1a). The patient reported that his distal clavicle was initially much more prominent, until he self-reduced it by manual pressure. Bilateral anterior posterior (AP) X-ray views of the clavicles were performed without (Fig. 2a) and with 10 kg weights attached to each wrist of the hanging arms (Fig. 2b). In addition, unweighted Alexander X-ray views were obtained from the healthy right (Fig. 3a) and affected left AC joint (Fig. 3b). X-rays confirmed the diagnosis of a Rockwood type V AC joint separation. A magnetic resonance imaging (MRI) of the left shoulder region confirmed a total rupture of the AC and the CC ligaments with a partial rupture of the trapezius muscle fascia (Fig. 4a) and a ruptured AC capsule (Fig. 4b).

Due to the patient’s wishes, the injury was treated conservatively using an AC joint brace (“acromion 2.0”, RO + TEN, Arcore, Italy), that provides a strap system, allowing depression of the clavicle with a broad shoulder pad, while simultaneously elevating the humerus towards the AC joint (Fig. 1b). The AC joint brace was adjusted by an orthopaedic technician one day after the injury and could afterwards be adjusted by the patient independently.

The AC joint brace reduced the AC joint as made evident by radiographic imaging taken one week after the injury (Fig. 2c).

The brace was worn day and night and only taken off for showering and physiotherapy sessions of 40 min/day. Physiotherapy began one week after the injury and was undertaken once or twice a week following a restrictive physiotherapy protocol (Table 1).

**Table 1:** Individual physiotherapy protocol followed by the patient

Week	Exercise
1	Absolute rest, no physiotherapy
2	Begin of physiotherapy with passive and active movements of the elbow joint
3 + 4	In addition to exercises of week 2, intensified physiotherapy with assisted passive abduction (90°), flexion (90°), external (45°) and internal rotation (85°) of the shoulder joint was added
5	In addition to exercises of week 2–4, pendular exercises for the shoulder joint without weights were added
6	In addition to exercises of week 2–5 simple active tasks were trained, including pressing down a door handle, lifting up an empty glass and removing books from a bookshelf
Post-brace period	Shoulder strengthening exercises, passive and active scapula mobilization, rotator cuff stretches and moderate weight-training were encouraged (no heavy weights for 8 weeks). To return to pre-injury strength and mobility, the patient increased exercise intensity gradually at this stage and did not have to further follow a strict protocol

During the first week after the injury, the patient reported reoccurring subluxations of the clavicle and a feeling of instability, whenever he took off the brace to shower. These subluxations gradually became less frequent and a more stable feeling of the AC joint was reported. From the second week after the injury onwards, no subluxations reoccurred and the patient reported a return of stability similar to the one before the injury.

The patient was able to return to work two weeks after the injury, still constantly wearing the AC joint brace and mainly performing one-handed computer tasks.

After six weeks of conservative treatment, the AC joint brace was taken off and bilateral AP X-ray views of the clavicles were obtained another three days later. The radiographs showed an aligned AC joint (Fig. 2d) with an anatomic reduction, comparable to the reduction, observed while the patient was still wearing the AC joint brace (Fig. 2c).

The patient reported of muscle soreness of the deltoid muscle and myofascial trigger points in the upper trapezius muscle after taking off the brace. Both symptoms subsided over the coming weeks. The active range of motion one week after removing the brace was 80° of abduction, 80° of flexion, 45° of external rotation and internal rotation to the T12 vertebra level. Within the third week after brace removal, the patient returned to the full pre-injury range of motion with an active abduction of 180°, flexion of 180°, external rotation of 90°, and internal rotation to the T7 vertebra level.

The patient was able to return to sports (running, gym, swimming), four weeks after the AC joint brace was taken off but was instructed not to lift heavy weights with the left upper extremity for another four weeks. Further, the cosmetic result was satisfying and showed no contour changes compared to the contralateral side. (Fig. 1c).

At the six-month follow-up, weighted X-ray views (10 kg) showed an AC joint that had healed in a Rockwood type II position with a slightly elevated clavicle compared to the uninjured contralateral side (Fig. 2e). The patient’s function was at a pre-injury level and especially no signs of scapula dyskinesia were evident.

The Subjective Shoulder Value at the six-month follow-up was 90 (range 0–100) [16], the Taft Score after the same period of time was 10 (range 0–12) [17] and the Constant Shoulder Score was 95 for the injured and 100 for the healthy side (range 0–100) [18].

## Discussion

### Efficacy and perspective of the proposed method

Until today there are no reports of devices that achieve clavicle depression and humeral elevation to realign the AC joint after high-grade AC joint separations. Cloth slings or shoulder immobilizers protect the shoulder from rotation and give support against gravity [15] yet they do not provide any form of active joint reduction.

We report for the first time a case of a successfully treated Rockwood type V injury with the aid of an AC joint brace that was able to achieve anatomic reduction of the AC joint without the risks of surgery. Reduction, cosmetic and functional outcomes were comparable to results that could previously only be achieved surgically [9, 19–21]. In a review from 2017, Van Bergen et al. stressed that surgeons should weigh a better cosmetic outcome against higher complication rates in surgically treated patients [9]. Results from this case report show that cosmetically and functionally favourable outcomes can both be achieved conservatively.

Initially the patient’s AC joint separation was classified as a Rockwood type III injury, following the evaluation of the unweighted bilateral AP X-ray view (Fig. 2a). Weighted X-ray views from the same visit unveiled a Rockwood type V injury (19 mm vs. 8 mm) (Fig. 2b). The literature is incoherent about the use of weights for the initial diagnosis of AC joint separations. Some authors argue that weights help to distinguish the severity of AC joint separations [22, 23] while others report a lack of efficacy of weighted radiographs [24]. Ibrahim et al. justified the use of weighted radiographs as an aid to unmask Rockwood type V injuries, which would otherwise be classified as Rockwood type III [23]. This constellation was confirmed by the present case. Six weeks after conservative treatment the patient showed no remaining radiographic signs of clavicle elevation, and six months post injury the patient’s clavicle had healed in a mildly elevated Rockwood type II position. This shows that a dynamic development of the injury is possible.

### Time of immobilization and rehab

As the recovery time with the AC joint brace was comparable to that of an abduction sling worn post-operatively after a surgical AC joint reconstruction, the restrictions of shoulder immobilization on everyday life are equal in both treatments [25]. The proposed rehab protocol, including a six-week immobilization is similar to the one suggested for surgically treated patients [9, 26].

### Costs

The literature reports costs for arthroscopic subacromial decompressions to be at $7246 per patient [27] and for arthroscopic rotator cuff repairs to be at $8985 per patient [28]. Costs for arthroscopic suture button AC joint reconstructions would likely be within the same price range [27]. The price of the herein reported AC joint brace lies below $100 and no overnight hospital stay is required. A shift from surgical to novel conservative treatments could drastically decrease the costly burden of AC joint separations on health care systems worldwide.

Although the patient was diagnosed with a Rockwood type V injury, non-operative reduction and stabilization were attempted using a dedicated AC joint brace. The radiographic follow-up of this “closed reduction and external fixation” approach was convincing with excellent functional, cosmetic and pain outcomes. Non-operative treatment of high-grade AC joint separations with a reduction brace might therefore be a treatment alternative in selected cases.
